# Promoter methylation of the *MGAT3* and *BACH2* genes correlates with the composition of the immunoglobulin G glycome in inflammatory bowel disease

**DOI:** 10.1186/s13148-018-0507-y

**Published:** 2018-06-04

**Authors:** Marija Klasić, Dora Markulin, Aleksandar Vojta, Ivana Samaržija, Ivan Biruš, Paula Dobrinić, Nicholas T. Ventham, Irena Trbojević-Akmačić, Mirna Šimurina, Jerko Štambuk, Genadij Razdorov, Nicholas A. Kennedy, Jack Satsangi, Ana M. Dias, Salome Pinho, Vito Annese, Anna Latiano, Renata D’Inca, IBD consortium, Daniel Kolarich, Manfred Wuhrer, Dermot P. B. McGovern, Iain K. Pemberton, Daniel I. R. Spencer, Daryl L. Fernandes, Rahul Kalla, Kate O’Leary, Alex T. Adams, Hazel Drummond, Elaine Nimmo, Ray Boyapati, David C. Wilson, Ray Doran, Igor Rudan, Paolo Lionetti, Natalia Manetti, Fabrizio Bossa, Paola Cantoro, Anna Kohn, Giancarlo Sturniolo, Silvio Danese, Marieke Pierik, David C. Wilson, Gordan Lauc, Vlatka Zoldoš

**Affiliations:** 1 Department of Biology, Division of Molecular Biology, Faculty of Science, University of Zagreb, Horvatovac 102a, 10000 Zagreb, Croatia; 2 Gastrointestinal Unit, Centre for Genomics and Molecular Medicine, University of Edinburgh, Edinburgh, EH4 6XU UK; 3 Genos Glycoscience Research Laboratory, Borongajska cesta 83h, 10000 Zagreb, Croatia; 4 Faculty of Pharmacy and Biochemistry, University of Zagreb, Zagreb, Croatia; 5 IBD Pharmacogenetics, University of Exeter, Exeter, UK; 6 Institute of Molecular Pathology and Immunology of the University of Porto (IPATIMUP), Porto, Portugal; 7 Department of Medical and Surgical Sciences, Division of Gastroenterology, University Hospital Careggi, Florence, Italy; 8 Department of Medical Sciences, Division of Gastroenterology, IRCCS-CSS Hospital, Viale Cappuccini, Rotondo, Italy; 9 Gastrointestinal Unit, University of Padua, Padua, Italy; 10 Translational Gastroenterology Unit, Nuffield Department of Medicine, University of Oxford, Oxford, UK

## Abstract

**Background:**

Many genome- and epigenome-wide association studies (GWAS and EWAS) and studies of promoter methylation of candidate genes for inflammatory bowel disease (IBD) have demonstrated significant associations between genetic and epigenetic changes and IBD. Independent GWA studies have identified genetic variants in the *BACH2*, *IL6ST*, *LAMB1*, *IKZF1*, and *MGAT3* loci to be associated with both IBD and immunoglobulin G (IgG) glycosylation.

**Methods:**

Using bisulfite pyrosequencing, we analyzed CpG methylation in promoter regions of these five genes from peripheral blood of several hundred IBD patients and healthy controls (HCs) from two independent cohorts, respectively.

**Results:**

We found significant differences in the methylation levels in the *MGAT3* and *BACH2* genes between both Crohn’s disease and ulcerative colitis when compared to HC. The same pattern of methylation changes was identified for both genes in CD19^+^ B cells isolated from the whole blood of a subset of the IBD patients. A correlation analysis was performed between the *MGAT3* and *BACH2* promoter methylation and individual IgG glycans, measured in the same individuals of the two large cohorts. *MGAT3* promoter methylation correlated significantly with galactosylation, sialylation, and bisecting GlcNAc on IgG of the same patients, suggesting that activity of the GnT-III enzyme, encoded by this gene, might be altered in IBD. The correlations between the *BACH2* promoter methylation and IgG glycans were less obvious, since *BACH2* is not a glycosyltransferase and therefore may affect IgG glycosylation only indirectly.

**Conclusions:**

Our results suggest that epigenetic deregulation of key glycosylation genes might lead to an increase in pro-inflammatory properties of IgG in IBD through a decrease in galactosylation and sialylation and an increase of bisecting GlcNAc on digalactosylated glycan structures. Finally, we showed that CpG methylation in the promoter of the *MGAT3* gene is altered in CD3^+^ T cells isolated from inflamed mucosa of patients with ulcerative colitis from a third smaller cohort, for which biopsies were available, suggesting a functional role of this glyco-gene in IBD pathogenesis.

**Electronic supplementary material:**

The online version of this article (10.1186/s13148-018-0507-y) contains supplementary material, which is available to authorized users.

## Background

Inflammatory bowel disease (IBD) is a chronic intestinal inflammatory condition classified in two major forms—Crohn’s disease (CD) and ulcerative colitis (UC)—which exhibit etiologically and clinically distinct features. Nowadays, IBD affects 2.5–3 million people in Europe and causes considerable morbidity [1]. Despite numerous clinical, genetic, and other experimental studies, our understanding of IBD development and progression remains incomplete.

It is generally accepted that IBD represents an aberrant immune response to gut microbiota in genetically susceptible individuals [2]. Genome-wide association studies (GWAS) have identified over 200 genetic susceptibility loci, the majority of which were associated with both forms of IBD in genome-wide meta-analysis [3–7]. However, common genetic variants account only for 8.2 and 13.1% heritability of UC and CD, respectively [7]. Interaction of an individual’s gut microbiome, immune system, genetic background, and environmental factors, such as smoking, diet, drugs, and physical activity [2, 8–10], makes IBD a complex etiopathogenic entity. The challenge is therefore to identify additional factors involved in the development and progression of this disease, especially given its rapidly increasing incidence. It is probable that epigenetics play a key role in the interactions between environmental, microbial, and genetic factors that participate in IBD development and progression. These include DNA methylation and histone modifications, as well as some other epigenetic mechanisms [11–13]; for a review, see [14, 15].

DNA methylation remains the most studied epigenetic modification, readily assayed in a large number of individuals/samples. Hypermethylation of gene promoters is generally associated with gene silencing, while promoter hypomethylation is associated with gene activation [16]. Environmentally changed DNA methylation pattern may contribute to the development of many complex diseases by mediating the interplay between external and internal factors and the gene expression [17–21]. There are also data to suggest that the aforementioned environmental modifiers of IBD can also affect DNA methylation [17–19, 22]. Epigenetic component of IBD has been addressed in many studies, mostly by whole genome methylation analysis performed on peripheral blood mononuclear cells (PBMCs) or mucosal tissue, revealing regions differentially methylated between the disease and healthy state, as well as between CD and UC [11–13, 23–26].

The majority of eukaryotic proteins are modified by addition of complex oligosaccharides (glycans) through the process of glycosylation. Therefore, glycans are an integral part of nearly all membrane and secreted proteins, including components of the immune system [27]. Aberrant protein glycosylation is implicated in virtually every human complex disease, including inflammation [28–31]. Previous studies have suggested that *N*-glycosylation of secreted and membrane proteins might be regulated epigenetically and that aberrant glycosylation profiles in disease can arise through aberrant epigenetics [32–38]. A comprehensive review about the role of protein glycosylation in IBD has been given recently [39]. *N*-glycosylation of serum-circulating proteins (such as the acute phase proteins; immunoglobulin G, IgG; and immunoglobulin A, IgA) or whole plasma *N*-glycome (i.e., *N*-glycans present on all plasma proteins) has been the focus of IBD biomarker discovery [36, 40–43]. In addition, our partners from IBD consortium and others established that altered glycosylation of IgG, which is a key effector of the humoral immune system, has a role in balancing inflammation at the systemic level [42–46].

GWA studies indicated associations of IBD with several loci involved in protein glycosylation [47, 48]. More recently, the first GWAS of IgG glycosylation identified 16 loci specifically associated with changes in IgG glycosylation [49]. Interestingly, five of these loci showed pleiotropy with IBD: *MGAT3*, a glyco-gene encoding for a glycosyltransferase, GnT-III; *LAMB1*, a member of transmembrane glycoprotein family of extracellular matrix; the *IL6ST*, a signal transducer shared by many cytokines; *IKZF1*; and *BACH2*, transcription factors involved in B cell differentiation, activation, and maturation. Only the *MGAT3* is a classical glyco-gene with a known function in IgG glycosylation, while the exact functional roles for other four GWAS hits in IgG glycosylation or IBD remain unknown.

In this study, we investigated promoter methylation differences in these five genes, associated with both IBD and IgG glycosylation, in peripheral whole blood of several hundred IBD patients from two independent cohorts. We also correlated promoter methylation data with IgG glycosylation data analyzed previously for the same IBD patients by our partners from the IBD consortium [43, 46, 50]. Peripheral blood was used for DNA methylation analysis and serum or plasma was used for glycan analysis, since one of our goals was the search for potential IBD biomarkers. As peripheral whole blood is a heterogeneous cell mixture with specific methylation pattern for each of the cell types [51], we also analyzed promoter methylation of our candidate genes in CD19^+^ B cells and CD3^+^ T cells isolated from peripheral blood mononuclear cells (PBMCs). B cells were of our particular interest since these cells produce IgG on their membrane and are precursors of plasma cells which secrete IgG. We have further explored if aberrant promoter methylation recorded in peripheral whole blood of IBD patients can be a proxy for epigenetic events occurring in the inflamed mucosa. To address this question, we analyzed DNA methylation from PBMCs, CD3^+^ T cells isolated from PBMCs, and CD3^+^ T cells isolated from inflamed colonic mucosa of UC patients from the third smaller cohort, for which biopsies were available.

## Methods

### Patient selection and ethics

Patients were recruited prospectively from Edinburgh, UK, and Florence, Italy, as a part of the IBD-BIOM project. The recruitment of patients from Edinburgh has been described elsewhere [13, 43]. Briefly, we recruited IBD patients prospectively as close as possible to the date of diagnosis from gastroenterology outpatient and endoscopy appointments between 2012 and 2015. We recruited symptomatic controls from gastroenterology clinics during the same period. In these individuals, we had excluded IBD and other organic bowel pathology following biochemical and/or endoscopic investigations. We recruited a further healthy volunteer cohort with no gastrointestinal symptoms. IBD patients were stratified by disease type (ulcerative colitis, UC, and Crohn’s disease, CD). Detailed genetic, phenotypic, and other data regarding IBD cases are given in Additional file 1: Tables S1 and S2. Florence cohort was collected through the network of the Italian Group for IBD (IG-IBD) since the beginning of 2001 and first described in 2005 [1] following an internal validation of phenotyping. Subsequently, longitudinal update has been performed on a yearly basis.

Ethical approvals were obtained from Tayside Committee on Medical Ethics B, and all patients and controls provided written, informed consent (LREC 06/S1101/16, LREC 2000/4/192).

### Florence recruitment details

IBD patients were prospectively recruited as close as possible to the date of diagnosis from gastroenterology outpatient and endoscopy appointments between years 2012 and 2015 in different tertiary referral centers in San Giovanni Rotondo, Rome, Rozzano (Milan), Padua, and Florence, Italy. Symptomatic controls were recruited in the same centers (gastroenterology clinics) during the same period. In these individuals, IBD and other organic bowel pathology were excluded by biochemical and/or endoscopic investigations. IBD patients were stratified by disease type (ulcerative colitis, UC, and Crohn’s disease, CD). Samples were obtained with the same methodology (see further) and centrally collected at San Giovanni Rotondo, Italy.

### Sample collection

We collected whole blood at the time of patient recruitment into 9-ml serum Z-clot activator tubes (Greiner), allowed them to clot at 4 °C for 60 min, and then centrifuged at 2500×*g* for 15 min. The serum was aliquoted off and stored at − 80 °C until further analysis.

A subset of patients and controls recruited in Edinburgh (Additional file 1: Table S3) underwent immunomagnetic cell separation to obtain CD19^+^ B cells. The methods have previously been detailed elsewhere [13]. Venepuncture using 9-ml K3 EDTA vacuette (Greiner) tubes was performed to obtain between of 18 and 36 ml of EDTA-buffered blood. An initial Ficoll (Ficoll-Paque, GE Healthcare, Bucks, UK) density gradient centrifugation was performed to obtain peripheral blood mononuclear cells. Cells labelled with antibody-coated microbeads (human CD8^+^ and CD19^+^ microbeads, 20 μl per 1 × 10^7^ cells) were immunomagnetic separated using the autoMACs Pro cell separator (Miltenyi, Germany). CD19^+^ separations were performed following an initial CD8^+^ depletion step. Nucleic acids were extracted using AllPrep (Qiagen, Hilden, Germany) according to the manufacturer’s guidance and stored at − 80 °C.

Colonic biopsies from controls and UC patients with inactive and active form of disease were mechanically dissociated to prepare single-cell suspensions using Hanks’ balanced salt solution modified medium, without calcium chloride and magnesium sulfate (HBSS) (Sigma), with penicillin/streptomycin and gentamicin. PBMCs were obtained by density gradient centrifugation using Lymphoprep. CD3^+^ T cells (from biopsies and blood) were magnetically sorted by using the EasySep™ Human T Cell Enrichment Kit (STEMCELL) following the manufacturer’s instructions. Following cell isolation, DNA extraction was performed using the Invisorb Spin Tissue Mini Kit (Stratec Molecular) following the manufacturer’s instructions.

### DNA methylation analysis

We analyzed promoter methylation of the candidate genes in the DNA from whole blood, as well as from the separated CD19^+^ B cells. In addition, for the *MGAT3*, which is a glycosyltransferase with direct and known function in IgG glycosylation [52], we analyzed promoter methylation—in DNA from PBMCs, CD3^+^ T cells isolated from PBMCs, and CD3^+^ T cells isolated from the colonic mucosa of healthy controls and UC patients (classified according to active and inactive form of the disease) of the third independent smaller subcohort collected by the Gastroenterology Department of Centro Hospitalar do Porto-Hospital de Santo António, Portugal (Additional file 1: Table S4). All specimens were subjected to histological examination and classification. All participants gave informed consent about all clinical procedures, and research protocols were approved by the ethics committee of CHP/HSA, Portugal (233/12(179-DEFI/177-CES).

For DNA methylation analysis, 500 ng of DNA from whole blood was bisulfite converted using EZ-96 DNA Methylation Gold kit (Zymo Research, Freiburg, Germany), and 100 ng of DNA from CD19^+^ B cells, PBMCs, and T cells was converted using EZ DNA Methylation Gold kit (Zymo Research, Freiburg, Germany) according to the manufacturer’s protocol. Two to six pyrosequencing assays were developed for promoter regions of each of the five candidate genes (*BACH2*, *MGAT3*, *IL6ST*, *IKZF1*, and *LAMB1*). The selection of analyzed CpG sites was random for assays 2–5 of the *MGAT3* gene. CpG sites within the *MGAT3* assay 1 were selected based on the GEO (Gene Expression Omnibus) database where methylation data were obtained using Illumina HumanMethylation450 BeadChip v1.1 technology. For the *BACH2* gene, assays were selected based on location of differentially methylated CpGs in different cell lines tested by ENCODE project, using Illumina HumanMethylation450 BeadChip v1.1 technology (a newer version, the Infinium MethylationEPIC 850K was not available at the time). We used traditional bisulfite-based protocols which cannot discriminate between 5-methylcytosine (5-mC) and 5-hydroxymethylcytosine (5-hmC) as oxidative bisulfite (oxBS-450K) method can [53]. However, recent studies have shown that global DNA hydroxymethylation is very low in blood cells [54, 55]. Furthermore, hydroxymethylation is significantly depleted from promotors and CpG islands, while enriched in the gene bodies [53, 56].

Based on the estimated statistical power, we did initial screening on 60 patients for each pyrosequencing assay, after which we excluded those genes (pyrosequencing assays) that did not show any statistically significant differences between IBD patients and healthy controls. Pyrosequencing assays for *LAMB1*, *IL6ST*, and *IKZF1* are shown in Additional file 2: Figure S1. We continued to analyze promoter methylation only in the *BACH2* and *MGAT3* genes. Specific regions were amplified using PyroMark PCR kit (Qiagen, Hilden, Germany). The cycling conditions for the *BACH2* gene were as follows: initial polymerase activation step for 15 min at 95 °C followed by 50 cycles of 30 s denaturation at 95 °C, primer annealing for 30 s at primer-specific temperatures (Additional file 1: Table S5), and 30 s at 72 °C, with final extension at 72 °C for 10 min. The cycling protocol used for amplification of the *MGAT3* gene fragments was described previously [35], with the annealing temperature adjusted to 55 °C for the fragment 1 performed on DNA from CD19^+^ B cells. For quantitative measurement of DNA methylation level at specific CpG sites, PCR-amplified bisulfite-converted DNA was sequenced using the PyroMark Q24 Advanced pyrosequencing system (Qiagen) according to the manufacturer’s recommendations. Sequences of PCR and pyrosequencing primers for the *BACH2* and the *MGAT3* genes are listed in Additional file 1: Table S5. EpiTect PCR Control DNA Set (methylated and unmethylated bisulfite-converted human DNA, Qiagen) was used as a control for PCR and pyrosequencing reactions.

### Statistical analysis

The nonparametric Mann-Whitney *U* test was used to compare the methylation status of CpG sites encompassed by the pyrosequencing assays in the *MGAT3* and *BACH2* genes between the two independent groups: HC compared to each of CD or UC. Significance threshold was set at *p* < 0.05 with additional Bonferroni correction for multiple testing. Given that age was our primary concern as a potential confounder, we visualized the age in the three groups (CD, UC, and HC) for the samples included in each analysis as violin plots (Additional file 3: Figure S2) and assured there was no significant difference between the age groups (*p* > 0.05) using the Mann-Whitney *U* test. This was done to assure the validity and strengthen the rationale for the selection of statistical methods.

For the data of the *MGAT3* promoter methylation from PBMCs, CD3^+^ T cells isolated from blood, and CD3^+^ T cells isolated from inflamed colonic mucosa (the Porto cohort), the Mann-Whitney *U* test was applied with Bonferroni correction accounting for 15 CpG sites.

### Glycan analysis

Glycans present on IgG were analyzed from serum of over 1000 IBD (UC and CD) patients and healthy controls in the Edinburgh cohort using ultra performance liquid chromatography (UPLC) [43, 50]. In the Florence cohort, plasma samples of 3500 IBD patients and healthy controls was used for analysis of IgG glycopeptides by liquid chromatography coupled to mass spectrometry (LC-MS) [46]. The data for IgG glycosylation analysis were used in this work for correlation analysis with promoter methylation data of *MGAT3* and *BACH2* genes, with matching samples from the very same patients and healthy controls.

### Isolation of IgG from blood plasma

IgG has been isolated from blood plasma by affinity chromatography using CIM Protein G 96-well plate (BIA Separations, Ajdovščina, Slovenia) and vacuum manifold (Pall Corporation, Port Washington, NY, USA) as previously described [57, 58]. In short, plasma samples (50–90 μl) were diluted with 1 × PBS, pH 7.4 in the ratio 1:7. All samples were filtered through 0.45 and 0.2-μm AcroPrep GHP filter plates (Pall Corporation) using vacuum manifold and immediately applied to preconditioned Protein G plate. After washing of the Protein G plate, IgG was eluted with 0.1 mol L^−1^ formic acid and immediately neutralized with ammonium bicarbonate to pH 7.0. Protein G plate was regenerated and stored at 4 °C.

### IgG glycosylation analysis using ultra-performance liquid chromatography

N-glycans from isolated IgG in the Edinburgh cohort were released with PNGase F after drying 300 μl of each IgG elution fraction, labeled with 2-aminobenzamide and excess of regents removed by clean-up using hydrophilic interaction liquid chromatography solid phase extraction (HILIC-SPE). Fluorescently labeled and purified *N*-glycans were separated by HILIC-UPLC using Acquity UPLC instrument (Waters, Milford, MA, USA) as previously described [43]. Samples were separated into 24 peaks [57], and the amount of *N*-glycans in each chromatographic peak was expressed as a percentage of total integrated area (% area).

### IgG glycosylation analysis using liquid chromatography coupled to mass spectrometry

In the Florence cohort, Fc-specific IgG glycopeptides were analyzed after IgG purification, overnight trypsin digestion at 37 °C, and reverse-phase purification on Chromabond C18 beads using vacuum manifold as described [46, 59]. Samples were analyzed using nanoliquid chromatography coupled to mass spectrometry (nanoLC-MS), on a nanoACQUITY UPLC system (Waters, Milford Massachusetts, USA) coupled to quadrupole-TOF-MS (Compact; Bruker Daltonics, Bremen, Germany) equipped with a sheath-flow ESI sprayer (capillary electrophoresis ESI-MS sprayer; Agilent Technologies, Santa Clara, USA) as previously described [46]. The nanoACQUITY UPLC system and the Bruker Compact Q-TOF-MS were operated under HyStar software version 3.2.

Data was processed as described previously [46, 60]. This resulted in the extraction of 16 IgG1, 16 IgG2/3, and 11 IgG4 glycoforms. The tryptic Fc-glycopeptides for IgG2 and IgG3 subclasses have identical peptide moieties in the Caucasian population and are therefore not distinguishable with this methodology. Annotation of the spectra was done based on accurate mass according to the relevant literature [40, 57].

### Correlation analysis

Methylation data for the *BACH2* (assay 2) and the *MGAT3* (assays 1 and 2) genes (obtained for the two large cohorts) were filtered according to the peak quality by rejecting peaks marked as “failed” by the pyrosequencing software. Average methylation across all assayed CpG sites was calculated for each pyrosequencing assay in each cohort. Methylation results for individual patients were matched with their corresponding glycan profiles. Sizes of datasets and patient classes obtained after including complete records (i.e., both methylation data and glycan profiles present) are shown in Additional file 1: Table S6. Individual glycan structures were represented as relative abundances and batch-corrected. Percentage of structures with bisecting *N*-acetylglucosamine was calculated for each cohort as a derived trait at this point. Glycan structures identified by each method were translated to Oxford notation, and only the 13 structures present in both the Edinburgh and Florence datasets were considered for correlation. We used IgG1 data from the Florence cohort, as this isoform was the most abundant. Three additional derived traits were calculated: ratios of FA2B to FA2, FA2BG1 to FA2G1, and FA2BG2 to FA2G2.

Pearson correlation between CpG methylation data and 17 glycan features (13 structures and 4 derived traits) was calculated. Significance threshold was set at *p* < 0.05 with additional Bonferroni correction for 17-fold multiple testing.

Methylation of assayed CpG sites in promoters of the *BACH2* and *MGAT3* genes was correlated with measured glycan structures. Pearson correlation coefficient along with the associated *p* value was calculated between average CpG methylation (for all genes/assays) and each measured IgG glycan structure. Calculation was done on pairwise complete observations. Only correlations with the *p* value below 0.01 were considered further. Next, correlation coefficients for all CpG assays were calculated, which was used to rank glycan structures according to regulation by the assayed region. Glycan structures with the strongest correlation (either positive or negative) to CpG methylation were then used to explain regulatory effects. All calculations and data visualizations were done in R language and environment for statistical computing (R Foundation for Statistical Computing, Vienna, Austria). Visualization of correlations was done using the R package “corrplot.”

## Results

### Promoter methylation of the candidate genes in whole blood and B cells of IBD patients

**Fig. 1:**
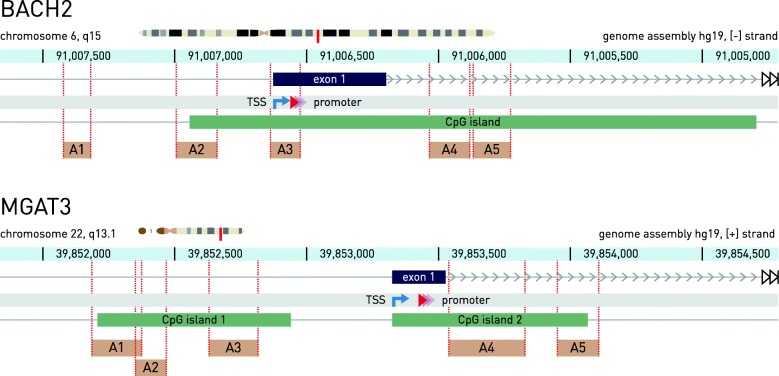
Positions of the *BACH2* and *MGAT3* genes in the human genome and relative positions of the fragments analyzed for methylation level (pyrosequencing assays) within these genes. For each pyrosequencing assay (A1–A5), the region amplified by PCR is shown. Positions of the genes on the chromosomes are shown using chromosome models (red vertical lines). Coordinates are relative to the hg19 human genome assembly. The genes are displayed in the direction corresponding to their reading frames. Annotations (CpG islands and pyrosequencing assays) are to scale. TSS transcription start site

In order to assess the level of methylation in CpG islands of the five candidate genes (*BACH2*, *MGAT3*, *IKZF1*, *LAMB1*, and *IL6ST*), associated with both IBD and IgG glycosylation by GWAS, we developed several pyrosequencing assays for each of the genes (Fig. 1 and Additional file 2: Figure S1). We performed initial screening of the pyrosequencing assays on 60 patients. Overall cytosine methylation levels were very low for *LAMB1* (average value per group < 8%), for *IL6ST* (< 3.5%), and for *IKZF1* (< 4%) in the assayed portion of their promoters; therefore, we could not identify differential methylation. We then excluded these genes from further analysis.

**Fig. 2:**
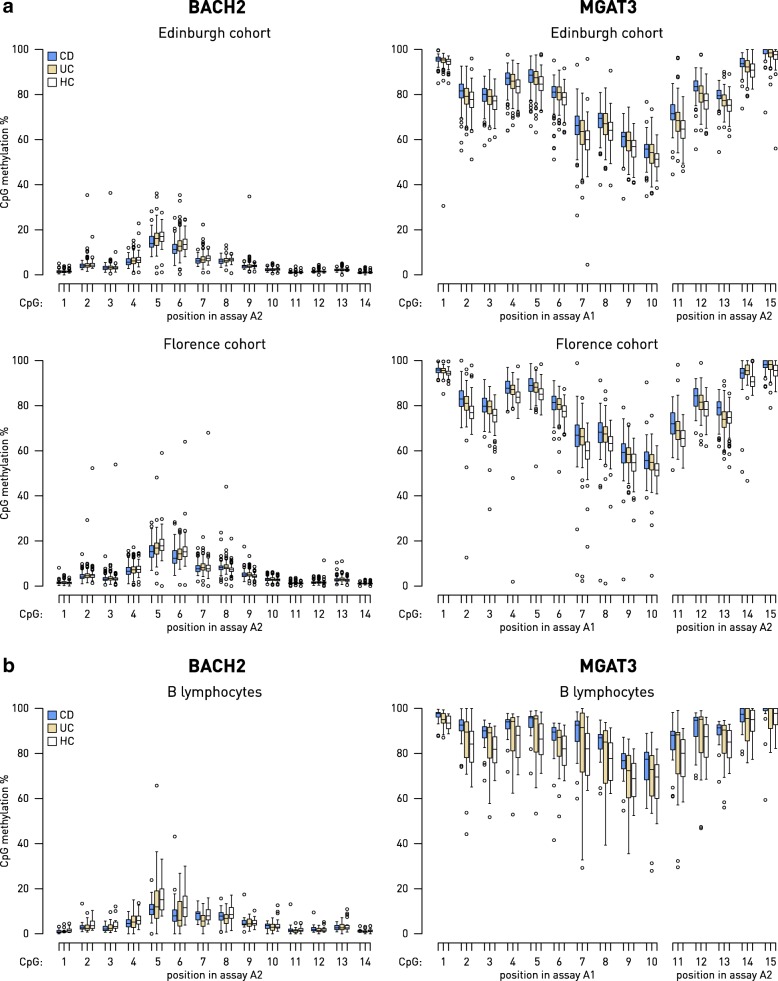
Box plot of CpG methylation in peripheral whole blood for the *BACH2* and *MGAT3* genes in the Edinburgh and Florence cohorts and in B cells from a subset of patients from Edinburgh cohort. Groups were compared using the Mann-Whitney *U* test with significance threshold of *p* = 0.05, corrected for multiple testing using the Bonferroni method. **a** Methylation levels were generally low in the assayed portion of the *BACH2* gene promoter, with significant differences between HC and CD methylation at CpG sites 4, 5, 6, and 8 (replicated in both cohorts). For the *MGAT3* gene, general methylation level was high, with all CpG sites showing a reproducibly significant difference between HC and both CD and UC, except for CpG sites 2, 13, and 15 for which reproducible significant differences were found only between HC and CD. **b** In B cells, isolated from PBMCs of a subset of the patients from the Edinburg cohort, differential methylation was found at the CpG position 5 of the *BACH2* gene (assay 2) between HC and CD, while for the *MGAT3* gene, differentially methylated were CpG sites 1–5, 12, and 13 between HC and CD. CD Crohn’s disease, UC ulcerative colitis, HC healthy controls

*MGAT3* and *BACH2* promoter methylation was analyzed in several hundred IBD patients and healthy controls from two independent cohorts (Additional file 1: Tables S1 and S2). In these genes, we analyzed methylation level at 47 CpGs covered by five pyrosequencing assays in the *BACH2* gene: 21 CpG sites were in the promoter region, 1 CpG site was in first exon, and 25 CpG sites were located in the first intron of the gene. A total of 32 CpG sites, covered by five pyrosequencing assays, was analyzed for *MGAT3*: 18 CpG sites were located in the promoter region and 14 CpG sites in the first intron (Fig. 1). Most of those CpG sites were located within CpG islands of the both genes. We found differential CpG methylation between IBD patients and HC within the assay A2, located at 213–368 bp upstream (relative to the gene orientation) of the TSS in the *BACH2* promoter and within the assays A1 and A2, located in the CpG island 1 of the *MGAT3* gene. The same pattern of differential methylation at these CpG sites was observed in whole blood of patients and HC from two large independent cohorts (Fig. 2). CpG methylation level was generally low (up to 20%) in the assayed portion of the *BACH2* promoter; however, significant differences between HC and CD methylation level were recorded at CpG sites 4, 5, 6, and 8 (Fig. 2a). For the assayed portion of the *MGAT3* promoter, general methylation level was high, with all CpG sites showing a reproducibly significant difference between HC and both CD and UC. CpG sites 2, 13, and 15 showed significant differences only for CD but not for UC. Direction of change was different for the two genes—differentially methylated CpG sites within the *BACH2* promoter were hypomethylated, while those for the *MGAT3* gene were hypermethylated in disease compared to healthy individuals.

These results were confirmed on CD19^+^ B cells isolated from peripheral whole blood of the independent, smaller patient sample from the Edinburgh cohort (67 samples). The CpG sites 1–5 and 12–13 in the *MGAT3* promoter were differentially methylated between CD and HC. Only CpG site 5 within the assay A2 of the *BACH2* gene showed change in the methylation level between HC and CD in CD19^+^ B cells (Fig. 2b). There were no differences in the methylation level of the same CpG sites within assayed fragments of the *BACH2* and *MGAT3* genes between UC and HC.

It is worth noting that the same pattern of CpG methylation differences was observed in PBMCs of the IBD patients and HC from both large independent cohorts, and most of the CpG sites within the assayed portion of the *MGAT3* promoter were also differentially methylated in CD19^+^ B cells from the subset of IBD patients from the Edinburgh cohort (Fig. 2a, b).

### Promoter methylation of the *MGAT3* gene in CD3^+^ T cells from PBMCs and inflamed colonic mucosa of UC patients

We included in our investigation biopsy samples of UC patients from an independent cohort from the Gastroenterology Department of Centro Hospitalar do Porto-Hospital de Santo António, Portugal. Given the technical challenges in obtaining DNA and RNA from a small number of purified cells from inflamed colonic mucosa, a subset of patients with active and inactive phase of UC was selected for methylation analysis from three sources: (1) PBMCs, (2) CD3^+^ T cells isolated from PBMCs, and (3) CD3^+^ T cells isolated from colonic mucosa (see also Additional file 1: Table S4).

**Fig. 3:**
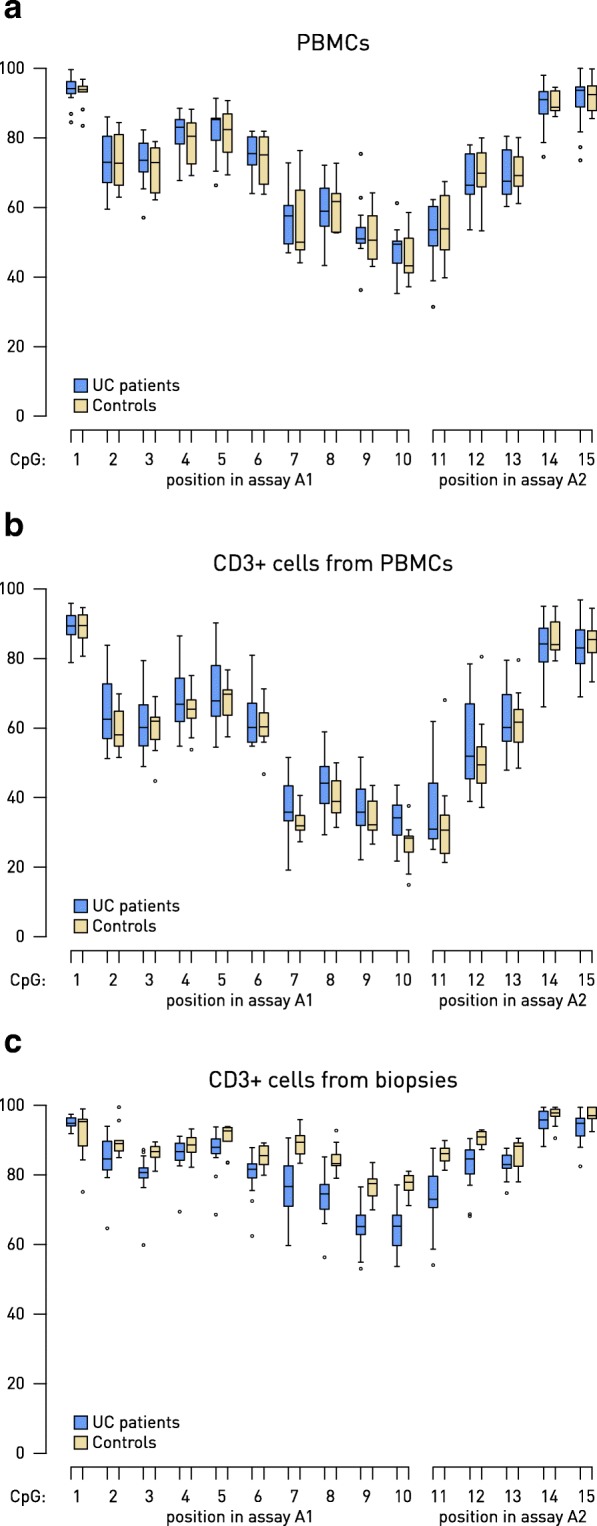
Box plot of CpG methylation level in the *MGAT3* gene promoter (assays A1 and A2) analyzed from PBMCs (**a**), CD3^+^ T cells isolated from PBMCs (**b**), and CD3^+^ T cells isolated from inflamed colonic mucosa (**c**) from the independent cohort of Porto. Changes between UC patients with active disease and HC were statistically significant only in CD3^+^ T cells isolated from inflamed colonic mucosa at CpG positions 3 and 7–12 (*p* < 0.05 after Bonferroni correction for 15 hypotheses). PBMC peripheral blood mononuclear cells, UC ulcerative colitis, HC healthy controls

Inter-individual variation of *MGAT3* methylation level measured from PBMCs and from CD3^+^ T cells isolated from PBMCs was quite large—it varied from 47 to 94% and from 26 to 90%, respectively. Therefore, we could not find any difference in CpG methylation level between UC patients and HC in assayed fragments of the *MGAT3* promoter, neither in PBMCs nor in CD3^+^ T cells isolated from PBMCs. However, we recorded a total of 7 (out of 15) differentially methylated CpG sites in CD3^+^ T cells isolated from colonic mucosa of UC patients with active disease compared with HC (Fig. 3). Overall, the methylation level of CpGs within assayed fragments of the *MGAT3* promoter was high in CD3^+^ T cells from healthy colonic mucosa (between 77 and 98%). When compared to inflamed mucosa of UC patients with active phase of the disease, the same CpG sites were hypomethylated, with the highest difference at the CpG position 10 (13.24%; *p* = 5.08 × 10^−5^; Fig. 3). In inactive UC, no significant differences could be found after Bonferroni correction for multiple testing.

It is worth noting that the methylation pattern in CD3^+^ T cells isolated from inflamed colonic mucosa differed from the methylation patterns in PBMCs and for CD3^+^ T cells isolated from PBMCs. The latter two were very similar and had much lower methylation levels than that measured for CD3^+^ T cells from inflamed colonic mucosa (Fig. 3). Also, *MGAT3* methylation level was increased in UC compared with HC when measured from PBMCs or CD3^+^ T cells isolated from PBMCs (hypermethylation), while it was decreased (hypomethylation) when measured from CD3^+^ T cells isolated from inflamed colonic mucosa in comparison with healthy mucosa.

### Correlation between the *MGAT3* and *BACH2* promoter methylation and IgG glycosylation

**Fig. 4:**
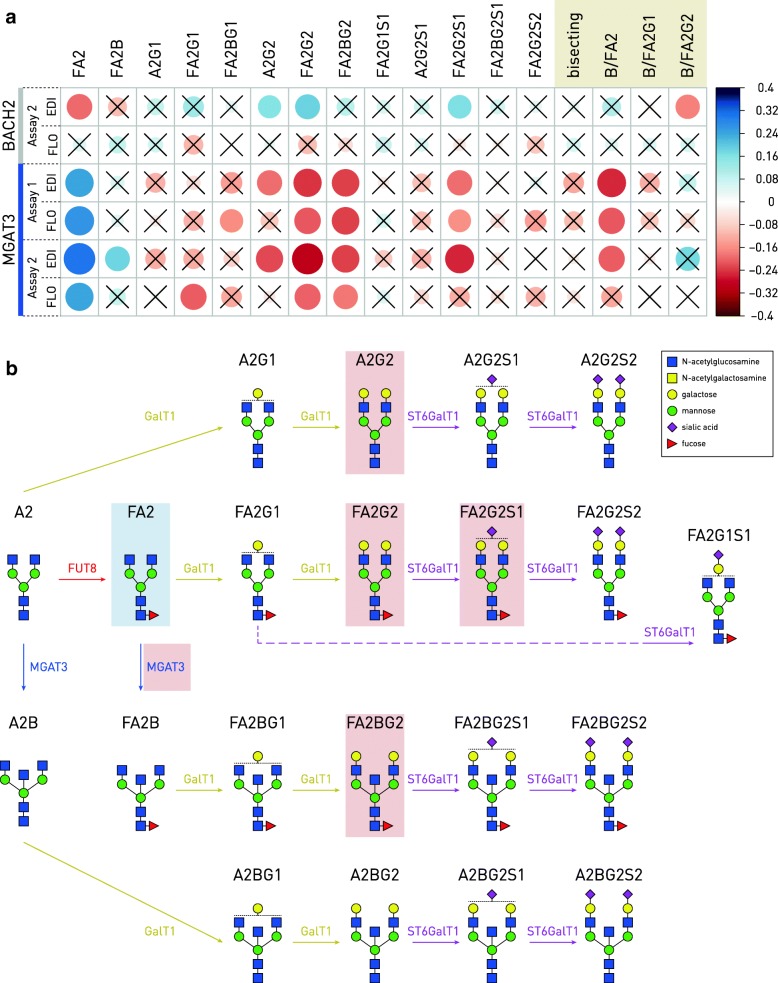
Correlations between CpG methylation in the *BACH2* and *MGAT3* gene promoters and glycan structures measured from the same individuals of the Edinburgh and Florence cohorts, mapped to the glycan biosynthesis pathways. **a** Correlation coefficients between average CpG methylation in the assayed gene promoter fragments and glycan structure percentages are shown as blue (positive) or red (negative correlation) circles with their size and shade proportional to the correlation coefficient. Correlations without statistical significance (*p* > 0.05 after Bonferroni correction for multiple testing) are crossed. Columns represent 13 individual glycan structures and four derived traits (beige box). EDI Edinburgh cohort, FLO Florence cohort, Bisecting, percentage of all structures with bisecting *N*-acetylglucosamine, B/FA2 ratio of FA2B to FA2 structures, B/FA2G1 ratio of FA2BG1 to FA2G1 structures, B/FA2G2 ratio of FA2BG2 to FA2G2 structures. **b** Glycan biosynthesis pathways with the glycan structures, labels, and the enzymes mapped to correlation results for the *MGAT3* gene. Light blue rectangles indicate positive, while light red rectangles indicate negative correlation between the glycan structures or traits and CpG methylation levels. Only correlations replicated across assays and/or cohorts are shown. The red rectangle around the MGAT3 enzyme reflects the negative correlation between CpG methylation and the derived trait B/FA2, which effectively measures enzyme activity at this step. MGAT3 *N*-acetilglucosaminyltransferase III (GnT-III), FUT8 fucosyltransferase 8, GalT1 galactosyltranserase 1, ST6GalT1 Beta-galactoside alpha-2,6-sialyltransferase 1

There were statistically significant correlations that replicated across assays and cohorts between the *MGAT3* promoter methylation and glycan structures FA2, FA2G2, FA2BG2, and FA2G2S1, as well as the derived trait of the ratio of FA2B to FA2 (Fig. 4a). All correlations except with FA2 were negative. No reproducible significant correlations could be found between *BACH2* promoter methylation and the glycan structures (Fig. 4a).

In order to infer a mechanistic pathway of the observed correlations, we mapped them to the glycan biosynthesis pathways (Fig. 4b). The ratio of bisecting glycans to FA2 was taken as an indicator of *MGAT3* (GnT-III) activity. This interpretation allowed us to infer lower GnT-III enzymatic activity when the promoter of the *MGAT3* gene was methylated. Increase in *MGAT3* promoter methylation correlated with a decrease in certain galactosylated and sialylated structures (Fig. 4b). In addition to the decreased levels of bisecting GlcNAc on non-galactosylated glycans (B/FA2), the most significant effect of the *MGAT3* promoter methylation on IgG glycome composition was a decrease of IgG galactosylation.

## Discussion

Results from this study strongly indicate that the *MGAT3* and *BACH2* genes play an important role in IBD pathogenesis and suggest a possible disease pathway mediated by the pro-inflammatory properties of IgG antibodies acquired by alterations in Fc glycosylation. Our recent study, performed on a large cohort of over 1000 IBD patients, reported a significant difference in IgG glycome composition in both UC and CD compared to healthy controls [43, 46]. We found a decrease in quantity of galacosylated glycans in both CD and UC, as well as a decrease in sialylated glycans and an increase of bisecting GlcNAc on digalactosylated glycan structures on IgG in CD. Indeed, alternative *N*-glycosylation of an IgG molecule influences its function—pro-inflammatory and anti-inflammatory activity depends on the glycans added on the Cy2 domain of its Fc region [29]. These glycans are of a biantennary complex type with or without bisecting GlcNAc, core fucose, galactose, and sialic acid residues [61]. Recently, this was confirmed in a large multi-centric study of IgG glycome in IBD [46]. Therefore, glycan changes observed on IgG in peripheral blood of UC and CD patients are obviously associated with increased inflammatory potential of IgG, suggesting functional relevance of IgG glycosylation for IBD.

Here, we propose a possible mechanism underlying the aberrant IgG glycosylation pattern observed in IBD [43, 46]. Out of five candidate genes analyzed in this work, the *MGAT3*, a glycosyltransferase which participates in synthesis of IgG glycans, and the *BACH2*, a transcription factor and a master regulator of a network of genes relevant for B cell integrity [62, 63], showed differential methylation in peripheral blood of both CD and UC patients when compared to healthy individuals. Even though we identified changes in methylation level for both UC and CD compared to HC, the differences were more pronounced for CD. This is concordant with other studies that explored either whole genome methylation or promoter methylation of candidate genes in IBD [11]. The extent of the change in IgG glycome composition was also consistently higher in CD than UC compared to HC [43, 46].

The protein encoded by the *MGAT3* gene (*N*-acetylglucosaminyltransferase III, GnT-III) is responsible for significant functional alteration of glycans on the Fc region of an IgG antibody. The GnT-III adds *N*-acetylglucosamine (GlcNAc) on β1,4-linked mannose in the three-mannose core of *N*-glycans, producing bisecting GlcNAc structures. In the same CD patients, who showed changed *MGAT3* promoter methylation level in peripheral blood cells, a significant increase in the percentage of bisecting GlcNAc on glycans of circulating IgG antibodies was recorded, too. The association of the *MGAT3* with both IgG *N*-glycosylation [49] and Crohn’s disease [4, 5] suggests that *N*-glycans with bisecting GlcNAc could be involved in CD pathogenesis through functional effect on IgG antibody.

Correlations between *BACH2* and *MGAT3* promoter methylation and glycan structures have given further insight into the changes of IgG glycosylation pattern mediated by those two genes (Fig. 4b). The *MGAT3* promoter methylation probably led to decreased GnT-III enzymatic activity, as revealed by negative correlation between methylation and total bisecting glycans to FA2 ratio. Namely, GnT-III adds a bisecting GlcNAc to FA2. A further proof is the positive correlation between the *MGAT3* promoter methylation and FA2, since it is not surprising that substrate accumulates when enzyme activity is decreased. More complex effects were observed on galactosylation and sialylation. The negative correlation between *MGAT3* methylation and galactosylation of both, glycans with (FA2BG2) and without bisecting GlcNAc (FA2G2), suggests that the effect of increased galactosylation is not caused only by steric effects of bisecting GlcNAc, but also through some indirect effects of *MGAT3* expression on galactosyltransferase activity. It seems as though galactosylation and sialylation are co-regulated with the addition of a bisecting GlcNAc catalyzed by GnT-III. Furthermore, Dekkers and co-workers recently reported that transfection of cells with *MGAT3* causes an increase of IgG galactosylation [64].

Much weaker correlation was observed between *BACH2* methylation and IgG glycosylation. This was expected since *BACH2* is not a glycosyltransferase and thus is not directly involved in glycan biosynthetic pathways. However, weak positive correlations with A2G2, FA2G2, and FA2G2S1 structures, which involve galactosylation and sialylation, were observed, as well as weak negative correlation with fucosylated bianntenary structure FA2. This is interesting because GWA studies associated *BACH2* with IgG galactosylation [49] as well as with various immune and inflammatory diseases including IBD [4, 5, 65–67] in which IgG acquires pro-inflammatory properties through decrease in galactosylation, sialylation, and fucosylation [43, 46]. Since *BACH2* is orchestrating a gene regulatory network in B cells [62], we believe that some glyco-genes are also regulated by this transcription factor. Indeed, our in silico analysis identified several glyco-genes, mostly galactosyltransferases (including *B4GALT1* and *B4GALT2*), to possess putative AP-1 and NFE2 binding sites for *BACH2* transcription factor [63], suggesting that these galactosyltransferases could be controlled by BACH2 (Additional file 1: Tables S7 and S8). Our present efforts are focused on functional studies with hope to reveal a more complete view of the *BACH2* role in IgG glycosylation.

Since DNA methylation pattern is tissue-specific, our goal was to ascertain if CpG methylation from blood could be a proxy for CpG methylation of the same candidate gene in the tissue where the inflammation is taking place. In fact, IBD is an immune-mediated disorder in which T cells are actively implicated in development of gut-mucosa inflammation [68]. Previous evidence has suggested that *N*-glycosylation of intestinal T cells is associated with UC pathogenesis and disease severity [50, 69]. Therefore, we analyzed *MGAT3* promoter methylation in CD3^+^ T cells isolated from PBMCs and from intestinal mucosa of UC patients with active and inactive form of the disease and compared with *MGAT3* promoter methylation from PBMCs of the same patients from the Porto cohort. We found 7 out of 15 differentially methylated CpG sites in CD3^+^ T cells isolated from colonic mucosa of UC patients with active form of the disease compared to CD3^+^ T cells from mucosa of healthy individuals. On the other hand, there were no differences in *MGAT3* promoter methylation between patients with either active or inactive form of UC and healthy controls neither in PBMCs nor in CD3^+^ T cells isolated from PBMCs. This could be due to high dispersion in the methylation level when measured from PBMCs (47–94%) and CD3^+^ T cells isolated from PBMCs (26–90%), probably due to small sample size and dispersion in age. Namely, cell composition changes across age in whole blood, and it can explain dispersion of CpG methylation level observed in our sample [70]. Considering much smaller dispersion in values of methylation level (65–97%) measured from CD3^+^ T cells from *lamina propria*, the differences could be used as a signature for inflammation.

## Conclusions

Taken together, our results suggest that the aberrant methylation observed in the *MGAT3* gene in CD3^+^ T cells from intestinal mucosa of UC patients, B cells from peripheral blood, and the whole peripheral blood in UC and CD patients is a possible mechanism underlying inflammation due to a change in the immune system—either through the change of glycans on Fc region of IgGs or by modulating the glycosylation profile of glycoproteins on intestinal T cells. Others [24] have shown that some of their candidate genes changed promoter methylation level in whole biopsies, while some of the genes showed changes only in some cell types of the heterogeneous cell population from the epithelial and non-epithelial cells, pointing out the importance of cell separation from mucosal biopsies. Interestingly, one of the genes that showed differential methylation in the non-epithelial fraction, representing immune and stromal cells, was *FUT7*, the fucosyltransferase involved in sialyl Lewis X synthesis, a ligand in selectin-mediated adhesion of leukocytes to activated endothelium. Furthermore, Dias and collaborators proposed a molecular mechanism in IBD involving another glyco-gene, the *MGAT5* (GnT-V), responsible for branching of *N*-glycans. They showed decreased expression of branched *N*-glycans on T cell receptor (TCR) of *lamina propria* associated with disease severity in patients with active UC [50]. Dysregulation of *N*-glycan branching on TCR contributes to a decreased threshold of T cell activation leading to a hyper-immune response which is a feature of UC patients. Taken together, our results and those of others suggest an important role of aberrant protein glycosylation (partly through epigenetic mechanisms) in IBD through dysregulation of the immune system. Also, in IBD diagnosis and treatment, it is important to find a non-invasive, specific, and clinically useful biomarkers in order to identify high-risk patients. Using *MGAT3* hypermethylation together with the glycan traits as markers from peripheral blood of IBD patients seems promising in the disease identification.

## Additional files

Additional file 1: **Supplementary Tables 1-8.** Demographics of IBD patients and healthy controls (1-4), PCR primers (5), number of samples per analysis (6) and in silico analysis of transcription factor binding sites in gene promoters (7, 8). (DOCX 70 kb)

Additional file 2: **Figure S1.** Position of the pyrosequencing assays for the genes *LAMB1*, *IL6ST*, and *IKZF1* in the genome relative to CpG islands, annotated promoters, and exons. (PDF 523 kb)

Additional file 3: **Figure S2.** Violin plots showing the age distribution in IBD patients (CD, UC) and healthy controls (HC). The groups were well matched by age, which was shown by Mann-Whitney *U* test: no significant differences between groups were found at the level *p* = 0.05. (PDF 704 kb)

## Abbreviations

CD: Crohn’s disease
EWAS: Epigenome-wide association study
GlcNAc: *N*-acetylglucosamine
GWAS: Genome-wide association study
HC: Healthy control
IBD: Inflammatory bowel disease
IgG: Immunoglobulin G
LC-MS: Liquid chromatography coupled to mass spectrometry
PBMCs: Peripheral blood mononuclear cells
UC: Ulcerative colitis
