# Supplementary material for: Promoter methylation of the MGAT3 and BACH2 genes correlates with the composition of the immunoglobulin G glycome in inflammatory bowel disease
Source: Clin Epigenetics. 2018 Jun 4;10:75. doi: 10.1186/s13148-018-0507-y (PMC5987481; doi:10.1186/s13148-018-0507-y)
Supplement: Supplementary file 1 — Supplementary Tables 1-8. Demographics of IBD patients and healthy controls (1-4), PCR primers (5), number of samples per analysis (6) and in silico analysis of transcription factor binding sites in gene promoters (7, 8). (DOCX 70 kb) [file 13148_2018_507_MOESM1_ESM.docx]

**Table S1.** Demographics of IBD patients and healthy controls from Edinburgh cohort analyzed for the *MGAT3* and *BACH2* promoter methylation in whole blood; MGAT3 and BACH2 assay = fragments in promoter of these genes analyzed for methylation level using pyrosequencing after bisulfite conversion; HC = healthy controls; UC = ulcerative colitis; CD = Crohn’s disease; N = number of analyzed individuals.

|  | **MGAT3 assay 1** | | | **MGAT3 assay 2** | | | **BACH2 assay 2** | | |
| --- | --- | --- | --- | --- | --- | --- | --- | --- | --- |
|  |  |  |  |  |  |  |  |  |  |
|  | **HC** | **UC** | **CD** | **HC** | **UC** | **CD** | **HC** | **UC** | **CD** |
|  | n=84 | n=237 | n=88 | n=72 | n=147 | n=62 | n=89 | n=263 | n=79 |
| **Sex** |  |  |  |  |  |  |  |  |  |
| Male | 31 | 95 | 30 | 32 | 63 | 21 | 34 | 107 | 30 |
| Female | 53 | 142 | 58 | 40 | 84 | 41 | 54 | 154 | 49 |
| Unknown |  |  |  |  |  |  | 1 | 2 |  |
| **Average age (years)** | 42.1 | 46.7 | 41.8 | 41.4 | 45.6 | 41.2 | 43 | 45.9 | 42.5 |
| **Ethnicity** |  |  |  |  |  |  |  |  |  |
| Asian | 2 | 4 | 1 | 1 | 3 |  | 2 | 5 |  |
| Black | 1 | 1 |  | 1 |  |  | 1 | 1 |  |
| Jewish | 1 | 1 |  | 1 | 1 |  | 1 | 1 |  |
| White | 72 | 225 | 80 | 64 | 138 | 56 | 78 | 247 | 73 |
| Other | 2 | 6 |  | 2 | 2 | 1 | 2 | 6 |  |
| Unknown | 6 |  | 7 | 3 | 3 | 5 | 5 | 3 | 6 |
| **Smoking status** |  |  |  |  |  |  |  |  |  |
| Current smoker |  | 27 | 30 |  | 21 | 22 |  | 32 | 26 |
| Ex-smoker |  | 86 | 4 |  | 48 | 4 |  | 87 | 4 |
| Non-smoker | 19 | 112 | 35 | 20 | 65 | 24 | 22 | 124 | 36 |
| Unknown | 65 | 12 | 19 | 52 | 13 | 12 | 67 | 20 | 13 |
| **Family history of IBD** |  |  |  |  |  |  |  |  |  |
| Yes |  | 46 | 10 |  | 24 | 7 | 4 | 51 | 12 |
| No |  | 189 | 65 |  | 119 | 46 | 81 | 207 | 56 |
| Unknown |  | 2 | 13 |  | 4 | 9 | 4 | 5 | 11 |
| **Family type** |  |  |  |  |  |  |  |  |  |
| CD |  | 13 | 7 |  | 5 | 4 | 2 | 15 | 8 |
| UC |  | 30 | 2 |  | 18 | 2 | 2 | 33 | 3 |
| Mixed |  | 2 | 1 |  |  | 1 |  | 2 | 1 |
| IBD unclassified |  | 1 |  |  | 1 |  |  | 1 |  |

**Table S2.** Demographics of IBD patients and healthy controls from Florence cohort analyzed for the *MGAT3* and *BACH2* promoter methylation in whole blood; MGAT3 and BACH2 assay = fragments in promoter of these genes analyzed for methylation level using pyrosequencing after bisulfite conversion; HC = healthy controls; UC = ulcerative colitis; CD = Crohn’s disease; N = number of analyzed individuals.

|  | **MGAT3 assay 1** | | | **MGAT3 assay 2** | | | **BACH2 assay 2** | | |
| --- | --- | --- | --- | --- | --- | --- | --- | --- | --- |
|  | **HC** | **UC** | **CD** | **HC** | **UC** | **CD** | **HC** | **UC** | **CD** |
|  | n=166 | n=143 | n=153 | n=139 | n=127 | n=98 | n=177 | n=169 | n=173 |
| **Sex** |  |  |  |  |  |  |  |  |  |
| Male | 135 | 93 | 88 | 118 | 85 | 51 | 143 | 103 | 100 |
| Female | 31 | 50 | 65 | 21 | 42 | 47 | 34 | 66 | 73 |
| **Average age (years)** | 40.6 | 46.2 | 43.6 | 40.2 | 46.1 | 44 | 40.3 | 46 | 44.1 |
| **Smoking status** |  |  |  |  |  |  |  |  |  |
| Curr. smoker |  | 17 | 60 |  | 15 | 38 |  | 20 | 69 |
| Ex-smoker |  | 56 | 31 |  | 50 | 20 |  | 61 | 32 |
| Non-smoker |  | 58 | 59 |  | 52 | 37 |  | 75 | 63 |
| Unknown |  | 12 | 3 |  | 10 | 3 |  | 13 | 9 |
| **Family history of IBD** |  |  |  |  |  |  |  |  |  |
| Yes |  | 12 | 11 |  | 12 | 7 |  | 13 | 12 |
| No |  | 127 | 139 |  | 112 | 88 |  | 151 | 154 |
| Unknown |  | 4 | 3 |  | 3 | 3 |  | 5 | 7 |

**Table S3.** Demographics of IBD patients and healthy controls from Edinburgh cohort analyzed for the *MGAT3* and *BACH2* promoter methylation in CD19+ B cells.

|  | **MGAT3 assay 1** | | | **MGAT3 assay 2** | | | **BACH2 assay 2** | | |
| --- | --- | --- | --- | --- | --- | --- | --- | --- | --- |
|  | **HC** | **UC** | **CD** | **HC** | **UC** | **CD** | **HC** | **UC** | **CD** |
|  | n=29 | n=18 | n=19 | n=29 | n=18 | n=20 | n=28 | n=19 | n=20 |
| **Sex** |  |  |  |  |  |  |  |  |  |
| Male | 16 | 12 | 10 | 16 | 13 | 11 | 15 | 13 | 11 |
| Female | 13 | 6 | 9 | 13 | 5 | 9 | 13 | 6 | 9 |
| **Average age (years)** | 34.9 | 39.1 | 34.7 | 34.9 | 38.8 | 34.2 | 34.8 | 39.8 | 34.2 |
| **Race** |  |  |  |  |  |  |  |  |  |
| Hispanic | 1 |  |  | 1 |  |  | 1 |  |  |
| Mixed race |  |  | 1 |  |  | 1 |  |  | 1 |
| White | 1 |  |  | 1 |  |  | 1 |  |  |
| White American |  |  | 1 |  |  | 1 |  |  | 1 |
| White Scottish | 5 | 2 | 1 | 5 | 2 | 1 | 5 | 2 | 1 |
| White European | 19 | 16 | 16 | 19 | 16 | 17 | 18 | 17 | 17 |
| Other | 1 |  |  | 1 |  |  | 1 |  |  |
| Unknown | 2 |  |  | 2 |  |  | 2 |  |  |
| **Smoking status** |  |  |  |  |  |  |  |  |  |
| Current smoker | 4 | 1 | 6 | 4 | 1 | 6 | 4 | 1 | 6 |
| Ex-smoker | 8 | 9 | 4 | 8 | 9 | 4 | 8 | 9 | 4 |
| Non-smoker | 16 | 8 | 9 | 16 | 8 | 10 | 15 | 9 | 10 |
| Unknown | 1 |  |  | 1 |  |  | 1 |  |  |

**Table S4.** Demographics of patients (with inactive and active phase of ulcerative colitis) and healthy controls from Porto cohort analyzed for the *MGAT3* promoter methylation in PBMCs, CD3^+^ T cells isolated from PBMCs, and CD3^+^ T cells isolated from inflamed colonic mucosa. (PBMCs = peripheral blood mononuclear cells; MGAT3 and BACH2 assay = fragments in promoter of these genes analyzed for methylation level using pyrosequencing after bisulfite conversion; HC = healthy controls; UC = ulcerative colitis; CD = Crohn’s disease; N = number of analyzed individuals)

|  | **MGAT3 assay 1** | | | **MGAT3 assay 2** | | |
| --- | --- | --- | --- | --- | --- | --- |
|  | **HC** | **inactive UC** | **active UC** | **HC** | **inactive UC** | **active UC** |
|  | n=11 | n=5 | n=19 | n=11 | n=5 | n=19 |
| **Sample number** |  |  |  |  |  |  |
| PBMCs | 9 | 5 | 13 | 10 | 5 | 13 |
| T cells from blood | 11 | 5 | 18 | 11 | 5 | 18 |
| T cells from tissue | 10 | 5 | 16 | 10 | 3 | 18 |
| **Average age (years)** | 56.2 | 56 | 51.5 | 56.2 | 56 | 51.5 |
| **Smoking status** |  |  |  |  |  |  |
| Smoker |  | 2 |  |  | 2 |  |
| Non-smoker |  |  | 7 |  |  | 7 |
| Unknown |  | 3 | 12 |  | 3 | 12 |
| **Family history of IBD** |  |  |  |  |  |  |
| Yes |  | 1 | 1 |  | 1 | 1 |
| No |  | 1 | 4 |  | 1 | 4 |
| Unknown |  | 3 | 14 |  | 3 | 14 |
| **Disease Treatment** |  |  |  |  |  |  |
| 5ASA |  | 4 | 9 |  | 4 | 9 |
| 5ASA and azathioprine |  |  | 2 |  |  | 2 |
| Azathioprine |  |  | 1 |  |  | 1 |
| Infliximab |  |  | 2 |  |  | 2 |
| Methotrexate |  | 1 |  |  | 1 |  |
| Methotrexate and adalimumab |  |  | 1 |  |  | 1 |
| No therapy |  |  | 2 |  |  | 2 |
| Unknown |  |  | 2 |  |  | 2 |
| **Biopsy region** |  |  |  |  |  |  |
| Ascending colon |  |  | 1 |  |  | 1 |
| Rectum |  | 1 | 4 |  | 1 | 4 |
| Rectum distal |  |  | 5 |  |  | 5 |
| Rectum proximal |  |  | 1 |  |  | 1 |
| Rectum/Sigmoid |  |  | 1 |  |  | 1 |
| Sigmoid | 1 | 4 | 3 | 1 | 4 | 3 |
| Sigmoid and rectum |  |  | 1 |  |  | 1 |
| Sigmoid distal | 1 |  |  | 1 |  |  |
| Transverse |  |  | 1 |  |  | 1 |
| Unknown | 9 |  | 2 | 9 |  | 2 |

**Table S5.** Primers used for PCR reactions and pyrosequencing on bisulfite converted DNA from Edinburgh (including CD19+ B cells), Florence and Porto cohort (assay = fragments in promoter of these genes analyzed for methylation level using pyrosequencing after bisulfite conversion; F = forward, R = reverse, SEQ = pyrosequencing).

| **Assay name** | **Primer name** | **Sequence (5'-3')** | **Purpose** | **Ta / °C** |
| --- | --- | --- | --- | --- |
| BACH2-1 | BACH2-F1 | TTTTGATAGGGTATTTATA | PCR | 44 |
|  | BACH2-R1 | [Btn]CAACTCCTACTACCTT |  |  |
|  | BACH2-SEQ1 | TTTTGATAGGGTATTTATA | SEQ |  |
| BACH2-2 | BACH2-F2 | GTGTTATATTAGTTTTTATTA | PCR | 50 |
|  | BACH2-R2 | [Btn]ACAACAAATCTCCCC |  |  |
|  | BACH2-SEQ2 | TTGTTATGTTTTTAATTTT | SEQ |  |
| BACH2-3 | BACH2-F3 | TTATTGTGAATGGGGA | PCR | 38 |
|  | BACH2-R3 | [Btn]ACTACTACTACTAAAAC |  |  |
|  | BACH2-SEQ3 | TTATTGTGAATGGGGA | SEQ |  |
| BACH2-4 | BACH2-F4 | GTTTTTATGGTATTTTTTAGG | PCR | 48 |
|  | BACH2-R4 | [Btn]TCCCTCTACTATTCCAAAA |  |  |
|  | BACH2-SEQ4 | ATGGTATTTTTTAGG | SEQ |  |
| BACH2-5 | BACH2-F5 | AGTAATAAGTTAGGTAT | PCR | 39 |
|  | BACH2-R5 | [Btn]ACCTAAAACTAAAATC |  |  |
|  | BACH2-SEQ5 | TAGGTATGGGGAGGG | SEQ |  |
| MGAT3-1 | MGAT3-F1 | GTTGGGATATAGAATAGGTAG | PCR | 54 (55 for B cells) |
|  | MGAT3-R1 | [Btn]ACCATTCCTCTCAAAACTCA |  |  |
|  | MGAT3-SEQ1 | GTTGGGATATAGAATAGGTAG | SEQ |  |
| MGAT3-2 | MGAT3-F2 | GTTTTTGAGTTTTGAGAGGAATGG | PCR | 60 |
|  | MGAT3-R2 | [Btn]ACCCTCTTAAACCTACTCTCCTAC |  |  |
|  | MGAT3-SEQ2 | GTTTTTGAGTTTTGAGAGGAATGG | SEQ |  |
| MGAT3-3 | MGAT3-F3 | AGTAGATATATAGATTTTGTAGA | PCR | 47 |
|  | MGAT3-R3 | [Btn]AATCTATACCTATATATACATAAAC |  |  |
|  | MGAT3-SEQ3 | AGTAGATATATAGATTTTGTAGA | SEQ |  |
| MGAT3-4 | MGAT3-F4 | GTTGTTGAGATTTAG | PCR | 51 |
|  | MGAT3-R4 | [Btn]CTAAAACTCTACCCTCC |  |  |
|  | MGAT3-SEQ4 | GGTGAGTTGATTT | SEQ |  |
| MGAT3-5 | MGAT3-F5 | GGGTTGGGGTGGGAGGTTTT | PCR | 60 |
|  | MGAT3-R5 | [Btn]CCCTCTCCACATTTACCTCTACCT |  |  |
|  | MGAT3-SEQ5 | GGGTTGGGGTGGGAGGTTTT | SEQ |  |

**Table S6.** Number of samples used for correlation analysis in Edinburgh and Florence cohorts stratified according to the type of the disease. (CD = Crohn’s disease; UC = ulcerative colitis; HC = healthy controls). A*n* = pyrosequencing assay *n*.

|  | **CD** | **UC** | **HC** | **total** |
| --- | --- | --- | --- | --- |
| *BACH2 A2 Edinburgh* | 74 | 260 | 78 | 412 |
| *BACH2 A2 Florence* | 147 | 143 | 163 | 453 |
| *MGAT3 A1 Edinburgh* | 88 | 237 | 84 | 409 |
| *MGAT3 A1 Florence* | 153 | 143 | 166 | 462 |
| *MGAT3 A2 Edinburgh* | 62 | 147 | 72 | 281 |
| *MGAT3 A2 Florence* | 98 | 127 | 139 | 364 |

**Table S7.** Number of predicted BACH2 and related transcription factor binding sites within the promoter region (defined as 2000 bp upstream from the transcription start site) of 9 selected genes connected with protein *N*‑glycosylation. Position-weight matrix (PWM) for each gene was obtained using the MotifDb R package (Shannon P and Richards M (2017). *MotifDb: An Annotated Collection of Protein-DNA Binding Sequence Motifs*. R package version 1.20.0.) and the HOMER database (http://homer.ucsd.edu/homer/motif/motifDatabase.html, accessed February 2, 2018). The PWMs used were for human AP1 (Hsapiens-JASPAR_CORE-AP1-MA0099.2), human NFE2 (Hsapiens-jolma2013-NFE2), mouse BACH1 (Mmusculus-JASPAR_2014-Bach1::Mafk-MA0591.1) and human BACH2 (Bach2(bZIP)/OCILy7-Bach2-ChIP-Seq(GSE44420)/Homer). The cut-off score for PWMs was 80%; regions with this or higher score were considered a match for the respective binding site.

|  | **gene** | **AP1** | **NFE2** | **mmBach1** | **Bach2** |
| --- | --- | --- | --- | --- | --- |
| 1 | BACH2 | 0 | 0 | 1 | 0 |
| 2 | LAMB1 | 1 | 0 | 0 | 1 |
| 3 | MGAT3 | 2 | 1 | 3 | 0 |
| 4 | SMARCB1 | 1 | 2 | 1 | 2 |
| 5 | DERL3 | 2 | 2 | 1 | 3 |
| 6 | SUV420H1 | 2 | 2 | 0 | 2 |
| 7 | B4GALT1 | 1 | 1 | 0 | 2 |
| 8 | ST6GAL1 | 5 | 5 | 0 | 0 |
| 9 | IKZF1 | 0 | 1 | 0 | 0 |

**Table S8.** Sequences and genome coordinates of the putative transcription factor binding sites listed in Table S7. Coordinates (start-end) are relative to the hg19 human genome assembly. chr = cgromosome; str = strand (+ or -).

|  | **gene** | **chr** | **start** | **end** | **str** | **sequence** | **motif** |
| --- | --- | --- | --- | --- | --- | --- | --- |
| 1 | BACH2 | chr6 | 91007894 | 91007908 | - | GCAGGGACTCTGCAG | mm-Bach1 |
| 2 | LAMB1 | chr7 | 107643969 | 107643975 | - | TGATTCA | AP1-core |
| 3 | LAMB1 | chr7 | 107643969 | 107643978 | - | TGGTGATTCA | Bach2 |
| 4 | LAMB1 | chr7 | 107643218 | 107643227 | - | TGCTTGCTCA | Bach2 |
| 5 | MGAT3 | chr22 | 39851394 | 39851400 | + | TGCCTCA | AP1-core |
| 6 | MGAT3 | chr22 | 39851526 | 39851532 | + | TGCCTCA | AP1-core |
| 7 | MGAT3 | chr22 | 39851559 | 39851569 | + | CATGAGCCACC | hs-NFE2 |
| 8 | MGAT3 | chr22 | 39852058 | 39852072 | + | AGGATCACTCTGCCT | mm-Bach1 |
| 9 | MGAT3 | chr22 | 39852140 | 39852154 | + | CGGATGGTTCAGCAA | mm-Bach1 |
| 10 | MGAT3 | chr22 | 39852636 | 39852650 | + | ACACAGACTCTGCAG | mm-Bach1 |
| 11 | SMARCB1 | chr22 | 24128990 | 24128996 | + | TGAGTCA | AP1-core |
| 12 | SMARCB1 | chr22 | 24127168 | 24127178 | + | AGTGGCTCATG | hs-NFE2 |
| 13 | SMARCB1 | chr22 | 24128714 | 24128724 | + | CAAGCCTCATC | hs-NFE2 |
| 14 | SMARCB1 | chr22 | 24128986 | 24129000 | + | TGCTTGAGTCAGGAC | mm-Bach1 |
| 15 | SMARCB1 | chr22 | 24127336 | 24127345 | + | GGCTGAGGCA | Bach2 |
| 16 | SMARCB1 | chr22 | 24128336 | 24128345 | + | GGCTGAGGCA | Bach2 |
| 17 | SMARCB1 | chr22 | 24128987 | 24128996 | + | GCTTGAGTCA | Bach2 |
| 18 | DERL3 | chr22 | 24182815 | 24182821 | - | TGCCTCA | AP1-core |
| 19 | DERL3 | chr22 | 24182344 | 24182350 | - | TGAGTCA | AP1-core |
| 20 | DERL3 | chr22 | 24182500 | 24182510 | - | TCTGTCTCATC | hs-NFE2 |
| 21 | DERL3 | chr22 | 24182342 | 24182352 | - | TTTGAGTCATG | hs-NFE2 |
| 22 | DERL3 | chr22 | 24181623 | 24181637 | - | TTAGTGAGCCAGCAC | mm-Bach1 |
| 23 | DERL3 | chr22 | 24182815 | 24182824 | - | TCCTGCCTCA | Bach2 |
| 24 | DERL3 | chr22 | 24182575 | 24182584 | - | TGCTGCATCA | Bach2 |
| 25 | DERL3 | chr22 | 24182376 | 24182385 | - | TGCTGGATCA | Bach2 |
| 26 | DERL3 | chr22 | 24182344 | 24182353 | - | TTTTGAGTCA | Bach2 |
| 27 | SUV420H1 | chr11 | 67959335 | 67959341 | - | TGAGTCA | AP1-core |
| 28 | SUV420H1 | chr11 | 67959159 | 67959165 | - | TGACTCT | AP1-core |
| 29 | SUV420H1 | chr11 | 67959333 | 67959343 | - | GATGAGTCAGT | hs-NFE2 |
| 30 | SUV420H1 | chr11 | 67958772 | 67958782 | - | AGTGGCTCATG | hs-NFE2 |
| 31 | SUV420H1 | chr11 | 67959335 | 67959344 | - | GGATGAGTCA | Bach2 |
| 32 | SUV420H1 | chr11 | 67959316 | 67959325 | - | TGGTGAGTAA | Bach2 |
| 33 | SUV420H1 | chr11 | 67958410 | 67958419 | - | TGCTCTGTCA | Bach2 |
| 34 | B4GALT1 | chr9 | 33168352 | 33168358 | - | TCACTCA | AP1-core |
| 35 | B4GALT1 | chr9 | 33168381 | 33168391 | - | CATGAGTCTTT | hs-NFE2 |
| 36 | B4GALT1 | chr9 | 33168352 | 33168361 | - | TGCTCACTCA | Bach2 |
| 37 | B4GALT1 | chr9 | 33167874 | 33167883 | - | GGCTGAGTTA | Bach2 |
| 38 | ST6GAL1 | chr3 | 186646320 | 186646326 | + | TGACTCA | AP1-core |
| 39 | ST6GAL1 | chr3 | 186646548 | 186646554 | + | TGACACA | AP1-core |
| 40 | ST6GAL1 | chr3 | 186646671 | 186646677 | + | TGACACA | AP1-core |
| 41 | ST6GAL1 | chr3 | 186647179 | 186647185 | + | TGCCTCA | AP1-core |
| 42 | ST6GAL1 | chr3 | 186647838 | 186647844 | + | TGCCTCA | AP1-core |
| 43 | ST6GAL1 | chr3 | 186646318 | 186646328 | + | ATTGACTCACA | hs-NFE2 |
| 44 | ST6GAL1 | chr3 | 186646546 | 186646556 | + | CCTGACACATG | hs-NFE2 |
| 45 | ST6GAL1 | chr3 | 186647177 | 186647187 | + | AATGCCTCAGG | hs-NFE2 |
| 46 | ST6GAL1 | chr3 | 186647829 | 186647839 | + | TAGGAGTCATG | hs-NFE2 |
| 47 | ST6GAL1 | chr3 | 186647836 | 186647846 | + | CATGCCTCAAT | hs-NFE2 |
| 48 | IKZF1 | chr7 | 50342811 | 50342821 | + | CATGACTTTTT | hs-NFE2 |
