# Supplementary material for: Promoter methylation of the MGAT3 and BACH2 genes correlates with the composition of the immunoglobulin G glycome in inflammatory bowel disease
Source: Clin Epigenetics. 2018 Jun 4;10:75. doi: 10.1186/s13148-018-0507-y (PMC5987481; doi:10.1186/s13148-018-0507-y)
Supplement: Supplementary file 2 — Figure S1. Position of the pyrosequencing assays for the genes LAMB1, IL6ST, and IKZF1 in the genome relative to CpG islands, annotated promoters, and exons. (PDF 523 kb) [file 13148_2018_507_MOESM2_ESM.pdf]

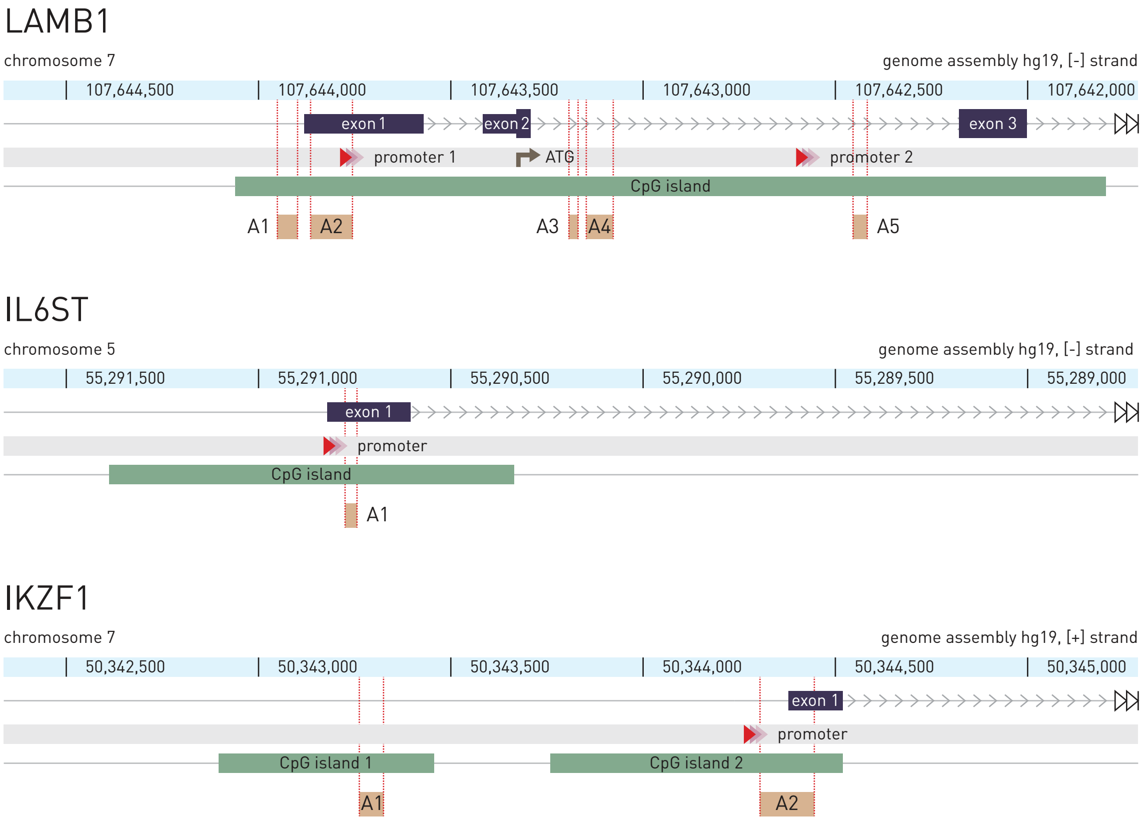

Figure S1. Position of the pyrosequencing assays for the genes *LAMB1*, *IL6ST* and *IKZF1* in the genome relative to CpG islands, annotated promoters and exons.
