# Supplementary material for: Promoter methylation of the MGAT3 and BACH2 genes correlates with the composition of the immunoglobulin G glycome in inflammatory bowel disease
Source: Clin Epigenetics. 2018 Jun 4;10:75. doi: 10.1186/s13148-018-0507-y (PMC5987481; doi:10.1186/s13148-018-0507-y)
Supplement: Supplementary file 3 — Figure S2. Violin plots showing the age distribution in IBD patients (CD, UC) and healthy controls (HC). The groups were well matched by age, which was shown by Mann-Whitney U test: no significant differences between groups were found at the level p = 0.05. (PDF 704 kb) [file 13148_2018_507_MOESM3_ESM.pdf]

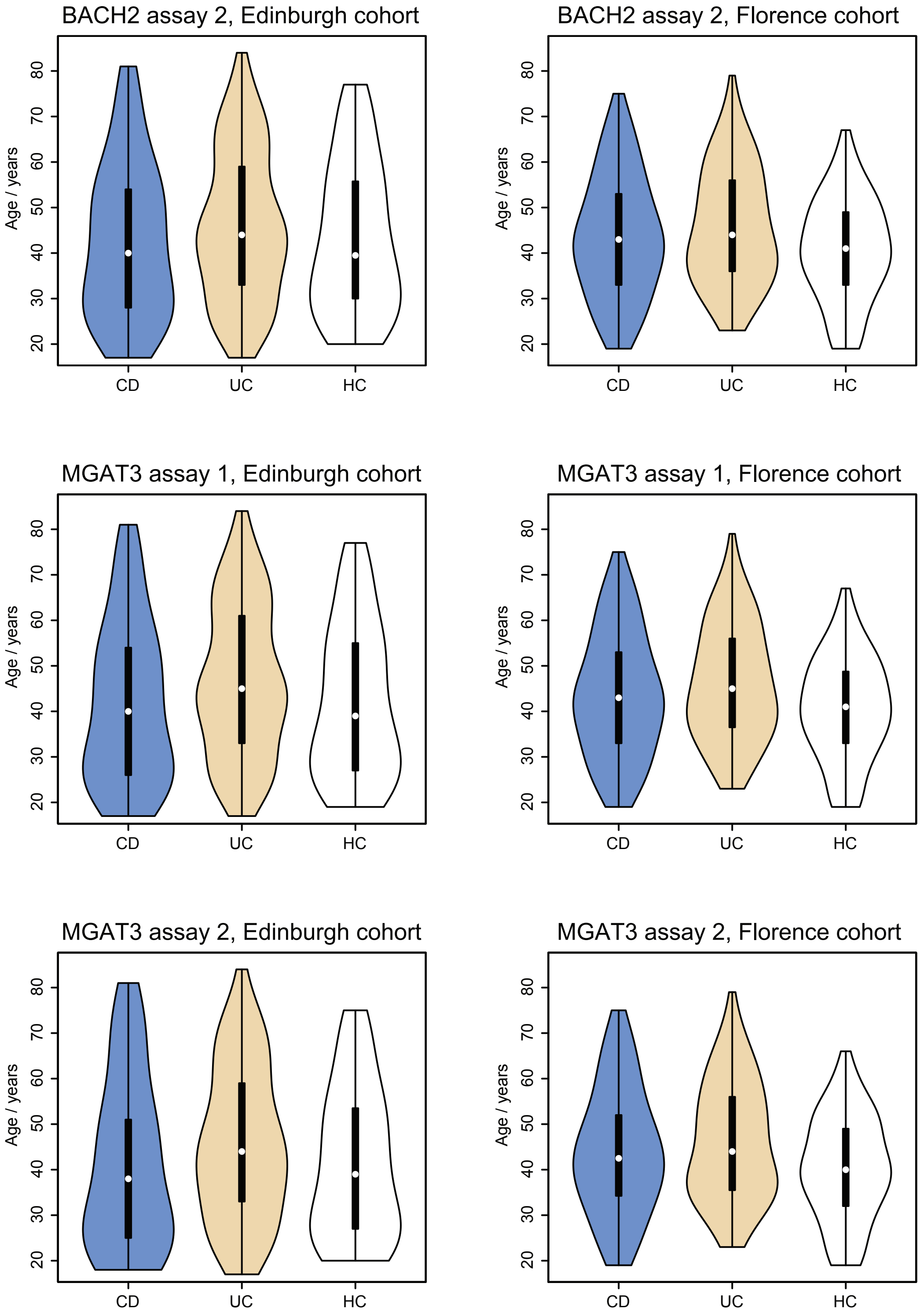

Figure S2. Violin plots showing the age distribution in IBD patients (CD, UC) and healthy controls (HC). The groups were well matched by age, which was shown by Mann-Whitney U test: no significant differences between groups were found at the level $p = 0.05$.
